# Fibroblast growth factor 21 associating with serotonin and dopamine in the cerebrospinal fluid predicts impulsivity in healthy subjects

**DOI:** 10.1186/s12868-021-00676-7

**Published:** 2021-11-20

**Authors:** Jinzhong Xu, Fenzan Wu, Yuying Li, Fan Wang, Wenhui Lin, Song Qian, Hui Li, Yuncao Fan, Huai Li, Lijing Chen, Haiyun Xu, Li Chen, Yanlong Liu, Xiaokun Li, Jue He

**Affiliations:** 1 Department of Clinical Pharmacy, Affiliated Wenling Hospital, Wenzhou Medical University, Wenling, Zhejiang China; 2 Laboratory of Translational Medicine, Affiliated Cixi Hospital, Wenzhou Medical University, Ningbo, Zhejiang China; 3 School of Pharmaceutical Sciences, Wenzhou Medical University, Wenzhou, Zhejiang China; 4 Beijing Hui-Long-Guan Hospital, Peking University, Beijing, China; 5 Key Laboratory of Psychosomatic Medicine, Inner Mongolia Medical University, Huhhot, China; 6 Central Laboratory, Affiliated Wenling Hospital, Wenzhou Medical University, Wenling, Zhejiang China; 7 The Criminal Science and Technology Department, Zhejiang Police College, Hangzhou, Zhejiang China; 8 Xinjiang Key Laboratory of Neurological Disorder Research, the Second Affiliated Hospital of Xinjiang Medical University, Urumqi, Xinjiang China; 9 The Affiliated Kangning Hospital, Wenzhou Medical University, Wenzhou, Zhejiang China; 10 School of Mental Health, Wenzhou Medical University, Wenzhou, Zhejiang China; 11 Institute of Aging, Key Laboratory of Alzheimer’s Disease of Zhejiang Province, Wenzhou Medical University, Wenzhou, Zhejiang China; 12 Institute of Neurological Disease, First Affiliated Hospital, Henan University, Kaifeng, Henan China

**Keywords:** Impulsivity, FGF21, Serotonin, Dopamine, Cerebrospinal fluid, Mental disorders

## Abstract

**Background:**

Impulsivity is more commonly reported in subjects with mental disorders compared to healthy subjects, suggesting a potential application of impulsivity in predicting impulsivity-related mental disorders. However, no biomarker of impulsivity available so far. This study explored the association between cerebrospinal fluid (CSF) fibroblast growth factor 21 (FGF21), a key hormonal mediator of the stress response, and impulsivity in healthy subjects.

**Methods:**

A total of 126 healthy persons subjected to surgery of anterior cruciate ligament were recruited in the present study. The impulsiveness of the subjects was evaluated by the Chinese version of the Barratt Impulsiveness Scale (BIS)-11 before surgery. CSF and blood samples of the subjects were collected before spinal anesthesia for surgery. The levels of FGF21, serotonin and dopamine in CSF and the level of FGF21 in blood of the subjects were measured by ELISA using commercial kits.

**Results:**

Negative correlations were found between BIS-11 total score and either FGF21, serotonin or dopamine in CSF. However, BIS-11 total score was not correlated with FGF21 in blood. In addition, FGF21 was positively correlated with serotonin and dopamine in CSF, respectively. Multivariable linear regression models indicated that the decrease of FGF21 level associating with the decrease of serotonin and dopamine level in CSF contributed to the higher impulsivity. Furthermore, receiver operating characteristic curve (ROC) analysis indicated an important role of CSF FGF21 predicting high impulsivity.

**Conclusions:**

FGF21, serotonin and dopamine in CSF associate with impulsivity in opposite directions. The decrease of CSF FGF21 is related to higher impulsivity, and indicate that CSF FGF21 may predict impulsivity in healthy subjects.

## Background

Impulsivity mainly characterized as “poorly anticipated and inappropriate hasty behaviors”, is usually relevant to negative emotions, undesirable consequences and even physical and mental health [1, 2]. As a personality trait, impulsivity is not only a part of normal behavior but also a potential shared pathological feature of mental disorders [2]. High levels of impulsivity are more common in subjects with conduct disorders, attention-deficit hyperactivity disorder (ADHD), disorders of personality, eating disorders, substance and alcohol abuse, psychotic disorders, bipolar disorders and dementia, suggesting the presence of a universal biological mechanism underlying the impulsivity in these mental and behavioral disorders [3–6]. Thus, it is very important to identify the subjects with high impulsivity tendency, and a biomarker of impulsivity will help to find, diagnose and treat the impulsivity-related mental disorders in the early stage in general population or currently healthy subjects. However, no reliable biomarker of impulsivity is available so far. In order to pursue the biomarkers of impulsivity, a potential biomarker in CSF and blood was investigated.

Fibroblast growth factor 21 (FGF21) functions as a stress hormone and a strong regulator of energy metabolism [7]. It regulates stress responses and modulates the functions of hypothalamic–pituitary–adrenal (HPA), which is closely associated with ADHD, suicide, and impulsive aggression [8–10]. In addition, FGF21 is used as a mood stabilizer because of its powerful neuroprotective effect [11]. Furthermore, increased FGF21 was associated with serotonin elevation in lipid metabolism [12], and the circulating FGF21 could regulate sucrose and alcohol preference in the presence of its co-receptor β-Klotho by reducing dopamine concentration in the nucleus accumbensin mice [13]. The potential neurochemical substances may be directly associated with the onset of impulsivity because similar neurochemical and brain activation patterns were found in high susceptibility subjects with impulsivity [14]. Magnetic resonance imaging data suggest that the striatal, prefrontal cortex and hippocampus play important roles in regulating impulsivity through activating neural circuitry interactions and neurochemical systems such as the dopamine, norepinephrine and serotonin systems [14–16].

In order to find out reliable biomarkers of impulsivity, the levels of FGF21 in CSF and blood of the subjects were measured and the correlations between FGF21 and impulsivity in CSF and blood were investigated to evaluate whether FGF21 involves in regulating impulsivity. Furthermore, the correlations among FGF21, serotonin, dopamine and impulsivity were investigated to evaluate whether FGF21’s regulating effect on impulsivity associates with its regulating effects on serotonin and dopamine in CSF in the present study.

## Methods

### Participants

A total of 126 subjects consisting of 93 males and 33 females were recruited at several Chinese hospitals in the current study. The included subjects were all scheduled to undergo surgery because of cruciate ligament injuries. CSF samples of the subjects were collected before spinal anesthesia. These subjects did not suffer from other trauma and received no medication. According to the criteria based on the Chinese version of Mini-International Neuropsychiatric Interview, subjects with mental disorders, neurological diseases, systemic diseases and central nervous system diseases were excluded from this study. Furthermore, according to the Diagnostic and Statistical Manual of Mental Disorders (4th edition), the subjects who had a diagnosis of drug abuse or dependence related to nicotine and alcohol were also excluded from the study. After approval by the Human Ethics Committee of Inner Mongolia Medical University, all participants were informed of the detail involving the right and interest in this study and then signed the written informed consent voluntarily. In addition, all methods were performed in accordance with the relevant guidelines and regulations by the Ethics approval and consent to participate. This study was performed in accordance with the Helsinki Declaration and International Ethical Guidelines for Biomedical Research Involving Human Subjects.

### Assessment of impulsivity

The Barratt Impulsiveness Scale (BIS)-11 was performed to evaluate the impulsivity of each participant before the surgery. A total of 30 items made up the BIS-11 scale, and the Chinese Version was involved in three topics: (1) non-planning impulsiveness (self-control and cognitive complexity), (2) motor impulsiveness (motor impulsiveness and perseverance), and (3) attentional impulsiveness (attention and cognitive instability) [17]. The validity, reliability, and predictive value of this version of the BIS-11 scale has been well evaluated and used widely to measure the impulsivity of subjects.

### CSF and blood collections

In a sterile environment, a licensed anesthetist performed the lumbar puncture using a spinal needle. The needle was inserted into the L3/L4 or L4/L5 intervertebral space when a patient was in the lateral decubitus position. A 5 mL CSF sample was drawn from each patient during lumbar puncture of spinal anesthesia before surgery. A 5 mL fasting blood sample was drawn after the patient was admitted to the hospital. Every 0.5 mL fraction of a sample was collected in tubes and frozen at −80 °C immediately until analysis.

### ELISA measurement of CSF and blood

The levels of FGF21, serotonin and dopamine in the CSF and the level of FGF21 in the blood were quantified by ELISA using commercial spectrophotometric measurement kits (Nanjing Jiancheng Bioengineering Institute, Nanjing, China) according to the manufacturer’s protocols [18]. All measurements were done in duplicate. Laboratory technicians were blinded to clinical data.

### Statistical analysis

The continuous variable was expressed as a mean ± standard deviation, and the categorical variable was expressed as frequencies. Partial correlation analysis was performed to evaluate the association between two variables with the year and education year as the covariate. A multivariable linear regression model was performed to evaluate the effect of biomarkers on impulsivity or the association between these biomarkers in CSF. Up to now, there is no specific demarcation value to define high impulsivity, but it is very important to distinguish and screen the population with high impulsivity, so we try to use the quantile commonly used in statistics to classify the impulsivity levels. BIS total scores more than the third quartile (75%) were used to indicate a high impulsivity risk compared with others in the current study. Receiver operating characteristic (ROC) curve analysis was performed to predict the subject with high impulsivity risk. A *P* value of less than 0.05 was regarded as statistically significant. All analyses were performed using SPSS 22.0 software (Statistical Package for the Social Sciences for Windows, Chicago, IL, USA and IBM SPSS Version 22.0).

## Results

### Demographic and clinical characteristics

A total of 93 males and 33 females were included in the present study, and the clinical characteristics of participants were shown in Table 1. The average age of participants was 30.46 ± 9.18 years, ranging from 17 to 50 years. The average years of education were 12.29 ± 3.95 years, ranging from 5 to 18 years. The average level of pain was 1.73 ± 0.83. The mean values of BIS scores were 24.38 ± 5.80 for non-planning, 26.01 ± 7.65 for motor, 24.60 ± 5.11 for attention, and 25.04 ± 5.38 for total scores. The levels of FGF21, serotonin and dopamine in CSF, and the level of FGF21 in blood were tested in all subjects. The mean value of serum FGF21 was 204.55 ± 9.25 pg/ml (from 190.47 to 264.06). The mean value of CSF FGF21 was 138.46 ± 35.08 pg/ml (from 73.05 to 202.05), CSF serotonin level was 312.66 ± 25.57 pg/ml (from 234.53 to 395.23), and CSF dopamine level was 89.59 ± 11.36 pg/ml (from 61.29 to 124.02) (Table 1). All surgeries for the cruciate ligament injury in the present study were the scheduled surgeries in patients whose CSF samples were collected before spinal anesthesia and whose general conditions and indications of blood test had recovered to normal before surgery. Therefore, CSF serotonin and dopamine levels obtained in the subjects before the scheduled surgery of the cruciate ligament injury in the present study and in normal subjects could be comparable, and dopamine and serotonin levels as shown in Table 1 in the present study might be equal to the baseline levels of dopamine and serotonin in the healthy subjects.

**Table 1:** Clinical characteristics of participants

The characteristic	Value
Female/male (number)	33/93
Age (years)	30.46 ± 9.18
Education years (years)	12.29 ± 3.95
BMI (kg/m^2^)	24.06 ± 3.83
Pain levels	1.73 ± 0.83
BIS Total Scores	25.04 ± 5.38
BIS nonPlanning	24.38 ± 5.80
BIS Motor	26.01 ± 7.65
BIS Attention	24.60 ± 5.11
Blood FGF21 Levels (pg/ml)	204.55 ± 9.25
CSF FGF21 Levels (pg/ml)	138.46 ± 35.08
CSF serotonin (pg/ml)	312.66 ± 25.57
CSF Dopamine (pg/ml)	89.59 ± 11.36

The continuous variable was expressed as a mean ± standard deviation after a normally distributed test. kg, kilogram; BMI, body mass index; m^2^, meter square; CSF, cerebrospinal fluid; FGF, fibroblast growth factor; BIS, Barratt Impulsiveness Scale

### CSF FGF21 was negatively associated with impulsivity

**Fig. 1:**
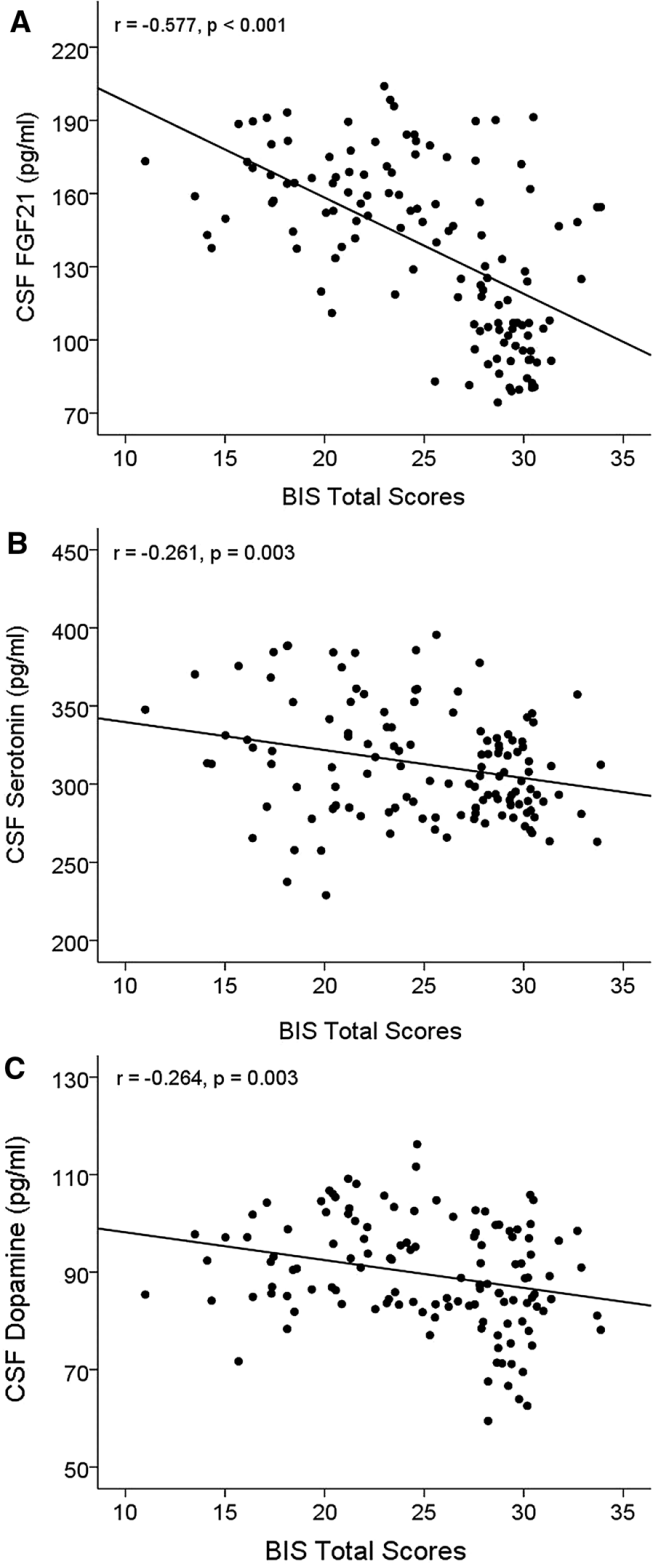
CSF biomarkers: FGF21 (**A**), serotonin (**B**) and dopamine (**C**) were negatively correlated with BIS total scores. The analysis was performed using the partial correlation with age and education years as covariates. Abbreviations: CSF, cerebrospinal fluid; FGF, fibroblast growth factor; BIS, Barratt Impulsiveness Scale

To evaluate the association between impulsivity and FGF21, a partial correlation was performed with age and education years as covariates. The result demonstrated that BIS total scores were significantly negatively correlated with CSF FGF21 (*r* =  − 0.577, *p* < 0.001) (Table 1, Fig. 1A), but there was no significant correlation between BIS total scores and serum FGF21 (*r* = 0.064, *p* = 0.479) (Table 2). A further analysis was performed to evaluate the association between the three dimensions of BIS and CSF FGF21: CSF FGF21was significantly negatively correlated with BIS non-planning (*r* = −0.399, *p* < 0.001), BIS motor (*r* = −0.634, *p* < 0.001), or BIS attention (*r* = −0.375, *p* < 0.001) (Table 2).

**Table 2:** The association between FGF21 levels and impulsivity

Variables	BIS total scores		BIS nonPlanning		BIS motor		BIS attention	
	*r*	*p*	*r*	*p*	*r*	*p*	*r*	*p*
Blood FGF21 Levels (pg/ml)	0.064	0.479	0.036	0. 916	0.083	0.358	0.023	0.798
CSF FGF21 Levels (pg/ml)	−0.577	< 0.001	−0.399	< 0.001	−0.634	< 0.001	−0.375	< 0.001

Partial correlations were used to analyze the relationship between FGF21 levels and impulsivity with the year and education year as covariates. FGF, fibroblast growth factor; BIS, Barratt Impulsiveness Scale

### CSF serotonin and dopamine were negatively associated with impulsivity

Previous studies have suggested that serotonin and dopamine are both implicated in impulsivity [19, 20]. The association between impulsivity and serotonin or dopamine in CSF was investigated. The result showed that CSF serotonin was significantly negatively correlated with BIS total scores (*r* =  − 0.261, *p* = 0.003) (Table 3, Fig. 1B), and further analysis showed that BIS Motor was significantly negatively correlated with CSF serotonin (*r* =  − 0.324, *p* < 0.001) (Table 3). CSF dopamine was also negatively correlated with BIS total scores (*r* =  − 0.264, *p* = 0.003) (Table 3, Fig. 1C), and further analysis showed that BIS Motor (*r* =  − 0.284, *p* < 0.001) and BIS Attention (*r* =  − 0.179, *p* = 0.046) were significantly negatively correlated with CSF serotonin (Table 3).

**Table 3:** The association between CSF serotonin, CSF Dopamine levels, and impulsivity

Variables	BIS total scores		BIS nonPlanning		BIS motor		BIS attention	
	*r*	*p*	*r*	*p*	*r*	*p*	*r*	*p*
CSF serotonin (pg/ml)	−0.261	0.003	−0.131	0.151	−0.324	< 0.001	−0.152	0.092
CSF Dopamine (pg/ml)	−0.264	0.003	−0.166	0.066	−0.284	< 0.001	−0.179	0.046

Partial correlations were used to analyze the relationship between impulsivity and CSF serotonin or dopamine with the year and education year as covariates. FGF, fibroblast growth factor; BIS, Barratt Impulsiveness Scale.

### FGF21 was positively associated with serotonin and dopamine in CSF

**Fig. 2:**
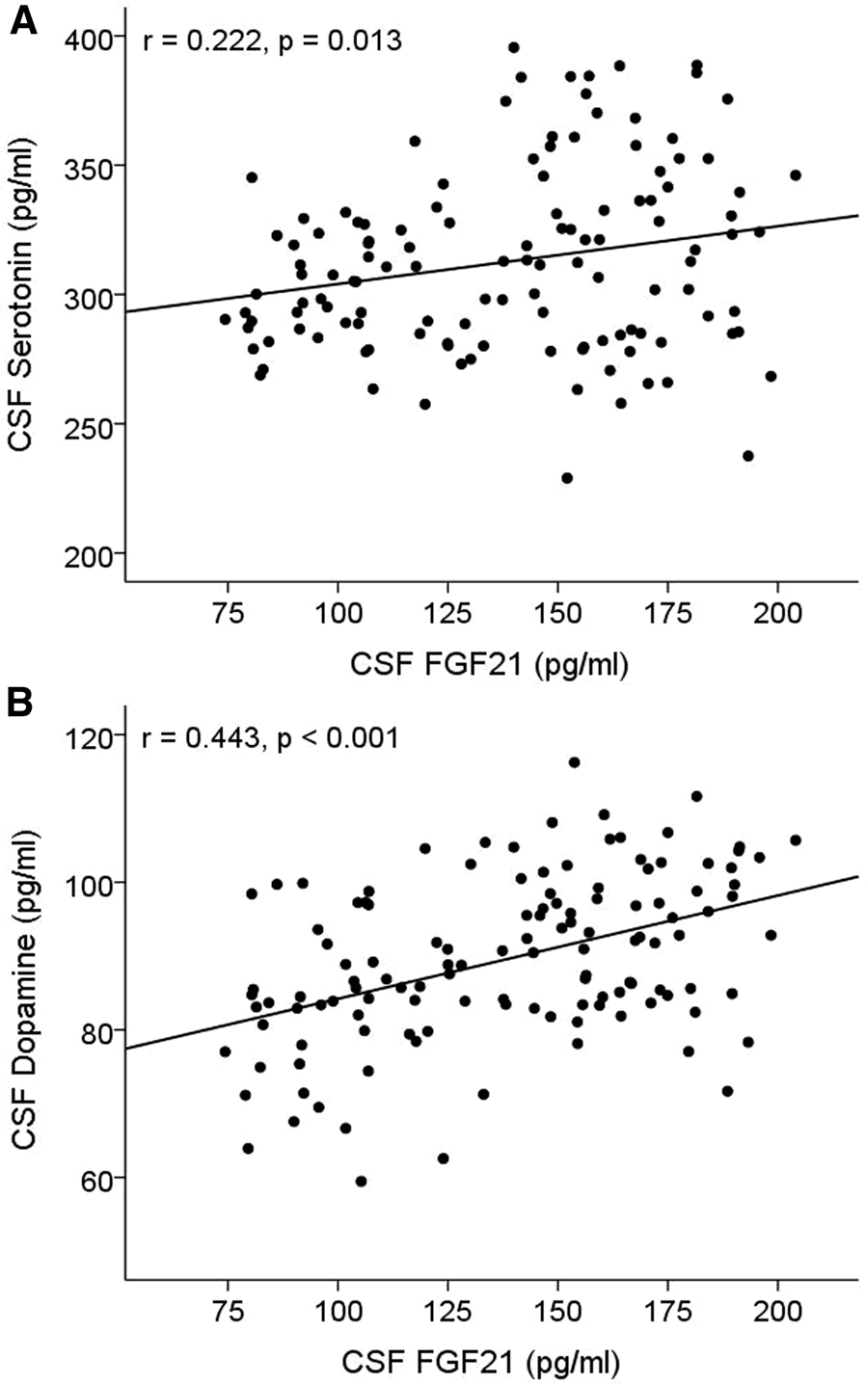
Serotonin (**A**) and dopamine (**B**) were positively correlated with FGF21 in CSF. The analysis was performed using the partial correlation with age and education years as covariates. Abbreviations: CSF, cerebrospinal fluid; FGF, fibroblast growth factor; BIS, Barratt Impulsiveness Scale

To explore the association of FGF21 with serotonin and dopamine in CSF, a partial correlation was performed with age and education years. FGF21 was significantly positively correlated with serotonin (*r* = 0.222, *p* = 0.013) (Fig. 2A) and dopamine (*r* = 0.443, *p* < 0.001) in CSF (Fig. 2B).

### The decrease of FGF21 contributed to the increase of impulsivity and the decrease of serotonin and dopamine in CSF

Multivariable linear regression analysis was performed to evaluate the effect of CSF FGF21 on impulsivity, and the results showed that the decrease of CSF FGF21 contributed to the increase of impulsivity in an unadjusted model (Model 1), which suggested that every 0.084 pg/ml decrease in CSF FGF21 may induce one unit increase of impulsivity. The results also showed that the decrease of CSF FGF21 contributed to the increase of impulsivity in Model 2 (adjusted for age and education years) and Model3 (adjusted for age, education years, BMI and pain levels) (Table 4).

**Table 4:** The multivariable linear regression analysis between CSF FGF21 and other variables

	BIS total scores	CSF serotonin (pg/ml)	CSF dopamine (pg/ml)
	β coefficient (95% CI)	β coefficient (95% CI)	β coefficient (95% CI)
CSF FGF21 (pg/ml)			
Model 1	−0.084 (−0.106; −0.062)^***^	0.226 (0.05; 0.402)^*^	0.143 (0.091; 0.195)^*^
Model 2	−0.084 (−0.106; −0.063)^***^	0.223 (0.048; 0.399) ^*^	0.140 (0.089; 0.191)^*^
Model 3	−0.088 (−0.111; −0.066)^***^	0.243 (0.057; 0.428) ^*^	0.130 (0.075; 0.184)^*^

Model 1: unadjusted

Model 2: adjusted for age and education years

Model 3: adjusted for age, education years, BMI and pain levels

^*^: *p* < 0.05,^***^: *p* < 0.001; FGF, fibroblast growth factor; BIS, Barratt Impulsiveness Scale; CI, confidence interval

The decrease of FGF21 contributed to the decrease of serotonin and dopamine levels in CSF in an unadjusted model (Model 1), which suggested that every 0.226 pg/ml decrease in FGF21 may induce one unit decrease of serotonin and that every 0.143 pg/ml decrease in FGF21 may induce one unit decrease of dopamine in CSF. The results also showed that the decrease of FGF21 contributed to the decrease of serotonin and dopamine in Model 2 (adjusted for age and education years) and Model 3 (adjusted for age, education years, BMI and pain levels) in CSF (Table 4).

### The decrease of serotonin and dopamine in CSF contributed to the increase of impulsivity

Multivariable linear regression analysis showed that the decrease of serotonin and dopamine in CSF contributed to the increase of impulsivity. In the unadjusted model (Model 1), every 0.034 pg/ml decrease in CSF serotonin may induce one unit increase of impulsivity, and every 0.105 pg/ml decrease in CSF dopamine may induce one unit increase of impulsivity. The results also showed that the decrease of serotonin and dopamine in CSF contributed to the increase of impulsivity in Model 2 (adjusted for age and education years) and Model 3 (adjusted for age, education years, BMI and pain levels) in CSF (Table 5).

**Table 5:** The multivariable linear regression analysis between impulsivity and biomarkers

Variables	BIS total scores		
	Model 1 β coefficient (95% CI)	Model 2 β coefficient (95% CI)	Model 3 β coefficient (95% CI)
CSF serotonin (pg/ml)	−0.034 (−0.059; −0.009)^**^	−0.038 (−0.063; −0.013)^**^	−0.037 (−0.063; −0.011)^**^
CSF Dopamine (pg/ml)	−0.105 (−0.184; −0.026)^**^	−0.122 (−0.202; −0.042)^**^	−0.116 (−0.200; −0.031)^**^

Model 1: unadjusted

Model 2: adjusted for age and education years

Model 3: adjusted for age, education years, BMI, and pain levels

^**^: *p* < 0.01; FGF, fibroblast growth factor; BIS, Barratt Impulsiveness Scale; CI, confidence interval

### CSF FGF21 predicted the participants with high impulsivity

**Fig. 3:**
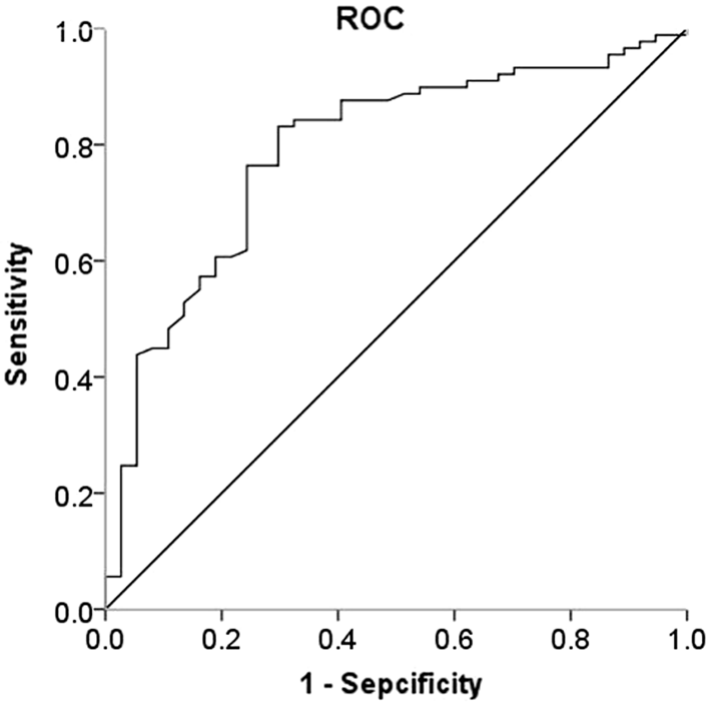
Receiver operating characteristic (ROC) curve analysis showed that the level of CSF FGF21 may be used to predict high impulsivity risk. The area under the ROC curve is 0.79 with a sensitivity of 70.27% and specificity of 83.15% (*P* < 0.001)

ROC curve analysis was performed to analyze the potential value of CSF FGF 21 for predicting the participants with high impulsivity and to investigate the cut‑off point. The optimal cut‑off point of CSF FGF21 for predicting subjects with high impulsivity was 117.34 pg/ml with a sensitivity of 70.27% and specificity of 83.15%, and the area under the curve (AUC) was 0.79 (*P* < 0.001; Fig. 3).

## Discussion

Impulsivity as a personality trait is a part of normal behavior, but an increased impulsivity is usually associated with many mental disorders. High level of impulsivity is more common in subjects with conduct disorders, ADHD, disorders of personality, eating disorders, substance and alcohol abuse, psychotic disorders, bipolar disorders and dementia, suggesting the presence of a universal biological mechanism underlying the impulsivity in these mental and behavioral disorders [3–6]. Therefore, a biomarker of impulsivity in blood or CSF of general population will help to identify the subjects with high impulsivity tendency, and to plan early treatment of mental disorders characterized by high impulsivity. However, so far there is no reliable biomarker of impulsivity found. Our current study is the first report showing that the increase of CSF FGF21 associates with the increase of impulsivity in the healthy subjects and indicating that CSF FGF21 may predict the risk of impulsivity in the currently healthy subjects. However, BIS-11 total score for impulsivity measurement was not correlated with the FGF21 level in blood in the present study. Our results have indicated that central dysfunctional FGF signaling for impulsivity may be independent to the level of circulating FGF level in blood. Therefore, the measurement of FGF21 in CSF would provide more valuable information for evaluating the risk of impulsivity than in blood.

FGF21 is considered an important pharmacological regulator and has the potential to serve as a biomarker for many diseases [21]. As a regulator of mood stabilizers, exogenous FGF-21 could protect aging neurons from glutamate challenge and might exert a strong neuroprotective effect through enhancing Akt-1 activation and glycogen synthase kinase (GSK)-3 inhibition in the central nervous system [11, 22]. Recombinant human FGF21 administration effectively improved obesity-induced cognitive dysfunction and anxiety-like behavior, and this suggested that FGF21 had a beneficial regulation on the central nervous system [23]. In the present study, the level of CSF FGF21 was negatively associated with BIS total scores and the multivariable linear regression analysis showed that the decrease of CSF FGF21 contributed to the increase of impulsivity. ROC analysis showed that CSF FGF21 had a relatively strong predictive value for the subjects with high impulsivity risk, and implied a high impulsivity tendency in subjects when CSF FGF21 was less than 117.34 pg/ml with a sensitivity of 70.27% and specificity of 83.15% for 0.79 area under the curve (AUC) (*P* < 0.001). Our results revealed the direct relationship between CSF FGF21 and impulsivity, and the decrease of FGF21 in CSF could predict impulsivity in healthy subjects. The current study demonstrated that CSF FGF21 may be an interesting and important biomarker used to evaluate the standard of impulsivity in the general population and to screen potential population of mental disorders related to impulsivity. However, the association between CSF FGF21 and impulsivity in the patients of mental disorders with impulsivity needs to be furtherly investigated.

The effect of decreased FGF21 on the increase of impulsivity may be mediated by CSF serotonin. Some early studies had evaluated the association between impulsivity and the limbic cortico-striatal systems, especially focusing on neurochemical substances such as serotonin, dopamine and noradrenaline [24, 25]. In the study using animal models, the decrease of central serotonin levels was found to be associated with the increases of behavioral disinhibition or impulsive action, and this suggested that serotonin may reflect impulsive tendencies [24]. Earlier studies had reported that the CSF serotonin metabolite (5-hydroxy indole acetic acid, 5-HIAA) was lower in impulsive offenders, and this suggested that lower 5-HIAA was associated with increased impulsivity [26, 27]. A recent neuroimaging (Positron Emission Computed Tomography and magnetic resonance imaging) study of measuring the global serotonin 4R binding for brain serotonin tonus revealed that low cerebral serotonin levels were associated with high levels of impulsive aggression in males [28]. Our data showed that CSF FGF21 was positively correlated with CSF serotonin, and the multivariable linear regression analysis showed that decreased CSF FGF21 contributed to the declined CSF serotonin. Therefore, we hypothesize that CSF FGF21 may affect impulsivity by protecting cerebral serotonin cells through Akt signaling pathway. However, further studies should be involved to demonstrate this hypothesis.

The effect of decreased FGF21 on the increase of impulsivity may be also mediated by CSF dopamine. FGF21 could affect dopamine signaling including changing the expression of dopamine-related genes, increasing dopamine transporter levels and directly reducing the levels of dopamine [13]. Dopaminergic mechanisms may involve in regulating impulsivity and interact with serotonin systems in the expression of impulsive behavior [29–31]. However, the conclusion about the role of dopamine in impulsivity was complex and contradictory in the studies of last decade. It has been reported that the patients who had high impulsivity combined with ADHD, had increased DA levels in synaptic and lower impulsivity after treatment with stimulant medications including methylphenidate and amphetamine [32, 33]. Contrasting with the results that the decreased D2/D3 auto receptor binding was correlated with higher impulsivity, higher impulsivity could be a partial result of stimulating striatal dopamine release [20]. Therefore, an inverted-U response of DA levels related to impulsivity was proposed [25, 34]. In the present study, CSF dopamine was negatively associated with impulsivity, and the multivariable linear regression analysis showed that the decreased CSF FGF21 contributed to the declined CSF dopamine. Since the mechanism of impulsivity is still unclear, the direct relationship and underline mechanism between CSF FGF21 and dopamine or serotonin in impulsivity need to be furtherly investigated. In addition, how FGF21 could affect dopamine signaling including changing the expression of dopamine-related genes should be investigated in in vitro and in vivo animal studies in the future.

The monoamine oxidase (MAO) enzyme is a key enzyme of serotonin and dopamine metabolisms. It has been reported that the MAO enzyme was closely associated with impulsivity, especially for low MAO B activity [35]. Chronic inflammation was also associated with impulsive tendencies [36, 37]. FGF21, as a hormone regulating stress responses, is well known for relieving numerous metabolic disorders related to inflammation, including metabolic syndrome and cardiovascular diseases [38]. A recent study found that FGF21 could improve depressive-like behavior induced by lipopolysaccharide (LPS)-induced by inhibiting the expression of proinflammatory cytokines [39]. Whether CSF FGF21 impacts impulsivity through mediating monoamine oxidase enzyme or inflammation remains to be furtherly studied.

Although the questionnaire measured impulsivity remains controversial, the BIS-11 scale is the most suitable method available now to assay the impulsivity of subjects [25]. Since we found that the decrease of CSF FGF21was associated with the increase of impulsivity, the level of CSF FGF21 may be used as an important index combining with the index of BIS-11 scale to assay the impulsivity of subjects in the general population. In addition, our present study showed that detecting CSF FGF21 to identify the subjects with high impulsivity tendency will help to find and treat mental disorders with high impulsivity in the early stage, and indicated that modification of CSF FGF21 by exogenous FGF-21 may be used as a new pharmacological therapy for mental disorders characterized by high impulsivity. Further future research should seek to clarify whether CSF FGF21 could be a biomarker of impulsivity for impulsivity-related mental disorders.

## Conclusions

In summary, FGF21, serotonin and dopamine in CSF associate with impulsivity in opposite directions, and the effect of CSF FGF21 on impulsivity may be related to the regulating effects of FGF21 on serotonin and dopamine in CSF. In addition, the present study demonstrated that CSF FGF21 may be a biomarker of impulsivity in the currently healthy subjects.

## Data Availability

The raw data supporting the conclusions of this article are available from the corresponding author on reasonable request, and the data are anonymized.

## Abbreviations

ADHD: Attention-deficit hyperactivity disorder
AUC: Area under the curve
BIS: Barratt Impulsiveness Scale
BMI: Body mass index
CI: Confidence interval
CSF: Cerebrospinal fluid
FGF: Fibroblast growth factor
GSK: Glycogen synthase kinase
HPA: Hypothalamic–pituitary–adrenal
kg: Kilogram
LPS: Lipopolysaccharide
m^2^: Meter square
MAO: Monoamine oxidase
ROC: Receiver operating characteristic curve
5-HIAA: 5-Hydroxy indole acetic acid
